# Cohort Profile Update: The Haematological Malignancy Research Network (HMRN) UK population-based cohorts

**DOI:** 10.1093/ije/dyab275

**Published:** 2022-02-04

**Authors:** Eve Roman, Eleanor Kane, Debra Howell, Maxine Lamb, Timothy Bagguley, Simon Crouch, Daniel Painter, Russell Patmore, Alexandra Smith

**Affiliations:** Epidemiology and Cancer Statistics Group, Department of Health Sciences, University of York, YO10 5DD, UK; Epidemiology and Cancer Statistics Group, Department of Health Sciences, University of York, YO10 5DD, UK; Epidemiology and Cancer Statistics Group, Department of Health Sciences, University of York, YO10 5DD, UK; Epidemiology and Cancer Statistics Group, Department of Health Sciences, University of York, YO10 5DD, UK; Epidemiology and Cancer Statistics Group, Department of Health Sciences, University of York, YO10 5DD, UK; Epidemiology and Cancer Statistics Group, Department of Health Sciences, University of York, YO10 5DD, UK; Epidemiology and Cancer Statistics Group, Department of Health Sciences, University of York, YO10 5DD, UK; Queens Centre for Oncology, Castle Hill Hospital, UK; Epidemiology and Cancer Statistics Group, Department of Health Sciences, University of York, YO10 5DD, UK

Key Features

- Established in 2004, the Haematological Malignancy Research Network is an ongoing population-based UK cohort that is currently tracking 38* *000 people diagnosed with a blood cancer or related disorder.
- Covering a population of ∼4* *million people (14 hospitals), each year ∼2500 people enter the cohort (irrespective of age or prognosis) on the day they are diagnosed.
- All diagnoses are made and coded using the World Health Organization’s latest International Diseases for Oncology classification by haematopathologists at a single fully integrated laboratory.
- HMRN operates on a legal basis that permits all patients to be tracked through local clinical systems and linked to national administrative databases (hospital episode statistics, cancers and deaths).
- Patients diagnosed between 2009 and 2015 (*n *=* *18* *127) have now been matched (year of birth, sex and residency in the study area) to 10 randomly selected controls from the national population-based National Health Service Central Register.
- The pseudonymized comparison cohort described in this update was designed to facilitate analyses requiring general-population background rates on co-morbidities and healthcare activity.

## The original cohort

Arising in blood and lymph-forming tissues, haematological malignancies (blood cancers) are the fourth most common cancer in men (after prostate, lung and bowel) and women (after breast, lung and bowel) in the UK and other economically developed countries.^1^^,^^2^ Traditionally classified by site [currently International Classification of Diseases version 10 (ICD-10)] according to whether cancer is first detected in blood (leukaemias), lymph nodes (lymphomas) or bone marrow (myelomas), the introduction of the World Health Organization (WHO)’s 2001 classification of tumours of haematopoietic and lymphoid tissues integrating genetic data with information on morphology, immunology and clinical parameters was paradigm-changing.^3^ Indeed, with diverse aetiologies, treatments and outcomes, >100 haematological cancer subtypes are currently recognized in the latest WHO International Diseases for Oncology classification, ICD-O-3.^4^ However, critically for epidemiology, although the WHO’s 2001 classification was rapidly adopted into clinical practice around the world, the radical nature of the changes posed significant challenges for population-based cancer registries; many struggling to capture the range and breadth of information required for implementation. Indeed, even now, descriptive data are often reported using the broad ICD-10 groupings of leukaemia, non-Hodgkin lymphoma, Hodgkin lymphoma and myeloma (e.g. ^5^^,^^6^).

Responding to the need for accurate population-based estimates on the occurrence, treatment and survival of the various disease subtypes, the Haematological Malignancy Research Network (HMRN) was established in 2004. Predicated on infrastructures within the UK’s National Health Service (NHS), where universal healthcare is freely provided on the basis of clinical need, full details of HMRN’s methods are presented in the original cohort profile^7^ and are available on the study website (www.hmrn.org). Briefly, operating on a similar legal basis to a cancer registry, all patients diagnosed with a haematological malignancy within HMRN’s catchment population of ∼4* *million are tracked through local clinical systems and linked to national administrative databases [deaths, cancer registrations and hospital episode statistics (HES)]. Served by 14 hospitals and a unified network of haematologists that operates across five multi-disciplinary teams (MDTs) and a network-wide paediatric MDT, clinical practice across the study area adheres to national guidelines. Importantly, within HMRN, all haematological cancers and related conditions are diagnosed and coded using the latest WHO ICD-O classification at the Haematological Malignancy Diagnostic Service (www.hmds.info)—a single integrated haematopathology laboratory that provides the national model for complex diagnostic services.^8^

**Figure 1:**
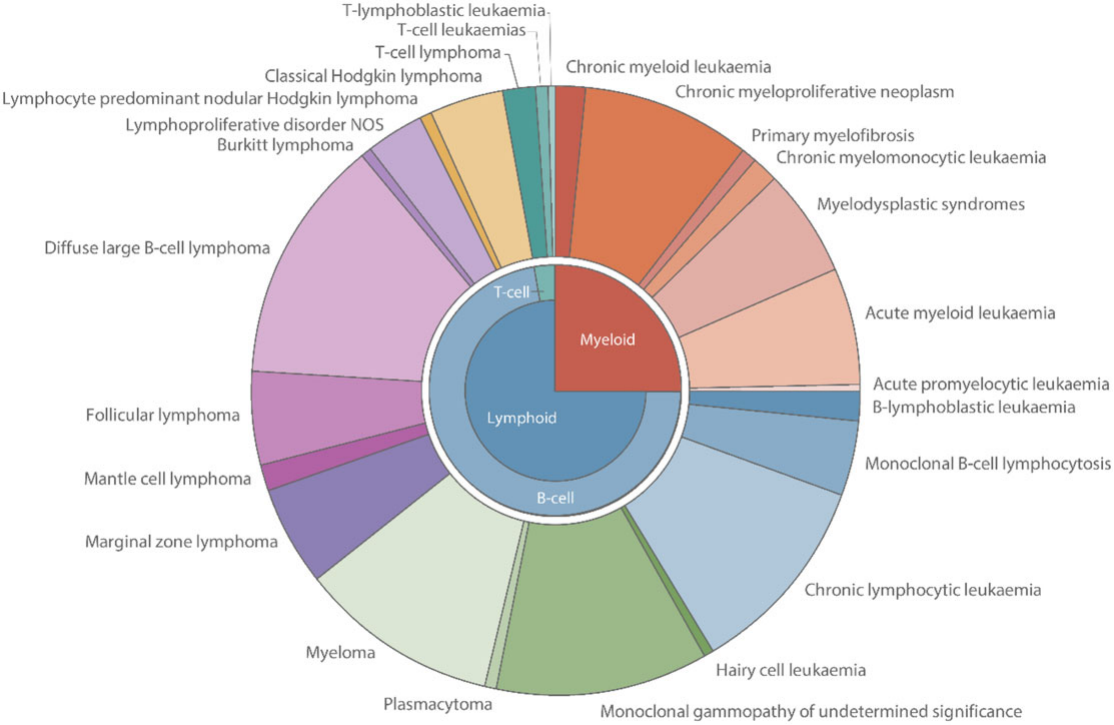
Diagnostic distribution of haematological malignancies and precursor conditions classified by ICD-O-3, Haematological Malignancy Research Network, 2004–2016

HMRN’s population has a socio-demographic profile that is broadly representative of the UK as a whole^7^ and all patients are included in the cohort, irrespective of their age, treatment intent, trial entry or management within the NHS or private sector. Providing generalizable information on clinically meaningful cancer subtypes (Figure 1), ∼38* *000 patients newly diagnosed in 2004–2019 with a haematological malignancy or related disorder have entered HMRN’s patient cohort, with a further 2500 new diagnoses being added each year. Disseminating findings via peer review^9–12^ and downloadable summary statistics from the website of the study (www.hmrn.org), HMRN’s descriptive data inform researchers, clinicians, patients and industry about the burden of disease across the population as a whole.

Embedding high-quality population-based epidemiological, clinical and biological research within the framework of NHS clinical practice, HMRN’s multifaceted cohorts have not, as far as we are aware, been replicated elsewhere in the world. Occupying a unique forefront position and with >50 peer-reviewed reports to date, HMRN’s size and sophistication allow researchers to apply the latest biological, statistical and health economic techniques to address a variety of research questions along the patient pathway. Since the first cohort profile was published, key publications have examined the clinical significance of cell-of-origin and molecular genetics,^13^^,^^14^ the impact of novel therapies in the general patient population,^15^ prediction models of cost and quality of life,^16^ routes to diagnosis and consequences of emergency presentation,^17–19^ experiences of patients with these cancers^20^^,^^21^ and end-of-life care.^22–24^

## What is the reason for the new data collection?

Importantly, although HMRN’s patient cohort can be used to answer many key research questions, epidemiological investigations requiring data on background levels of co-morbidity and/or healthcare activity in the general population were initially limited to making basic comparisons with other published series or national data.^9–11^^,^^25^ Accordingly, with a view to facilitating research requiring more detailed information about the health of unaffected individuals, HMRN investigators have now incorporated a general-population comparison cohort into the study design; and it is the utility of the new data generated by this addition that is the focus of the present report.

## What will be the new areas of research?

Linked to the same nationwide administrative databases as the patient cohort (deaths, cancer registrations and HES), the comparison cohort comprises an age- and sex-matched pseudonymized cohort of unaffected individuals. This allows the health of people diagnosed with a haematological malignancy or related condition to be compared with unaffected people in the general population, enabling the quantification of risks both before and after the detection of a haematological disorder. Hence, the new research areas largely comprise investigations examining associations with other cancers and co-morbidities that are managed/treated in secondary care, as well as short- and long-term trends in levels of hospital activity.

## Who is in the cohort?

The comparison cohort contains individuals who were matched on sex and year of birth to patients who were diagnosed with a haematological malignancy within the HMRN region between 2009 and 2015. In order to construct the comparison cohort, the date of birth, date of diagnosis, sex, NHS number, and HMRN study number of the 18* *127 patients diagnosed in 2009–2015 were uploaded to NHS Digital (https://digital.nhs.uk/), where they were matched to 10 randomly selected individuals from the national population-based NHS Central Register. All comparison cohort members (*n* = 181* *270) were resident in the HMRN study region when their corresponding case was diagnosed (month/year) and all were assigned the same HMRN diagnosis date (pseudo-diagnosis date) and a study number that linked to their matched case.

## What has been measured?

**Figure 2:**
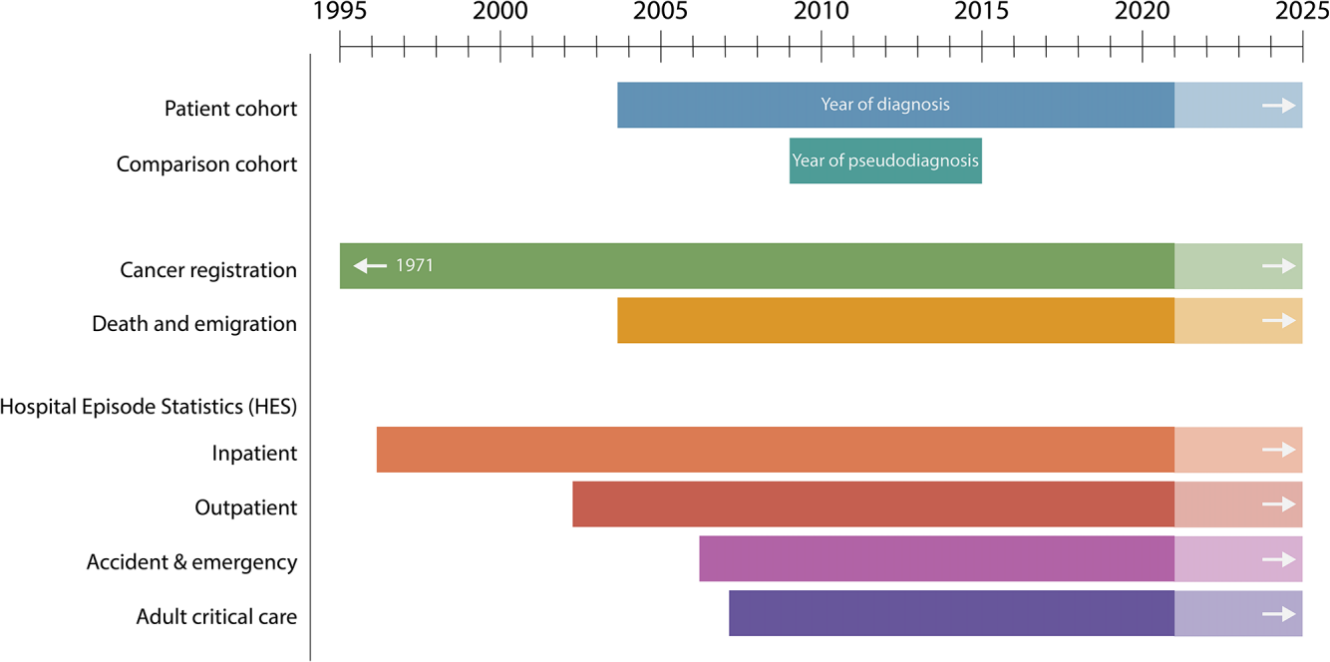
National data availability: Haematological Malignancy Research Network patient cohort and comparison cohort

Providing new data for analysis, HMRN’s comparison cohort was linked by NHS Digital to the same nationwide administrative databases as the patient cohort (deaths, cancer registrations and HES). The time periods covered by the various data sets, which are updated annually by NHS Digital, are shown in Figure 2.

The key data domains and fields available for analysis in both the patient cohort and the pseudonymized comparator cohort are shown in Table 1. A number of additional variables, listed in the HES data dictionaries (https://digital.nhs.uk/data-and-information/data-tools-and-services/data-services/hospital-episode-statistics), are not listed here because they are rarely recorded and cannot be used for analytical purposes; e.g. although fields for diagnostic details are provided in outpatient HES, the data are usually missing. In this context, it is also worth noting that although routinely compiled administrative data sets such as HES are increasingly used for research purposes, both in the UK and elsewhere in the world, this is not necessarily the main purpose for which the data were originally recorded in the hospital setting. For example, much of the information on inpatient discharge summaries (e.g. morbidities that are subsequently ICD-10-coded) is recorded by clinicians for patient-care purposes, whereas other inpatient HES details are primarily recorded for payment purposes (e.g. healthcare resource group codes).

**Table 1:** Data available for comparative analysis

Data	Description
Socio-demographics	Sex; year of birth; date of diagnosis/pseudo-diagnosis; Index of Multiple Deprivation income-domain at the time of diagnosis/pseudo-diagnosis
Death notifications	Date and causes of death (ICD-10)
Emigrations	Date of embarkation
Cancer registrations	Date of diagnosis; date of registration; topography (ICD revisions 7–10); morphology (ICD-O revisions 1–3)
Hospital episode statistics (HES)	
Inpatient	Date of admission; date of discharge; dates and types of procedures (maximum 24); conditions at discharge (ICD-10, maximum 20); consultant specialties involved; source of referral; discharge destination
Outpatient	Date of appointment and attendance flag; types of procedures (maximum 24); consultant specialties involved
Accident & Emergency	Date and reason for attendance; investigations and treatments; source of referral
Adult critical care (linked to inpatient)	Date of admission and discharge from critical care; organs supported; days of support for cardiovascular, respiratory, dermatological, gastrointestinal, liver, neurological and renal organs; source of referral; discharge destination

## What has it found? Key findings and publications

Thus far, data from the comparison cohort have been used to investigate a number of relationships between haematological malignancies diagnosed in 2009–2015 and other illnesses and procedures.^26–28^ First, since immune dysregulation plays a pivotal role in the development of mature B-cell malignancies and autoimmune conditions, outpatient HES data were used to examine the relationship between mature B-cell malignancies and preceding rheumatological disorders. In this context, it is important to note that the UK’s National Institute for Health and Clinical Care Excellence (NICE) specifies that conditions such as rheumatoid arthritis and Sjögren’s syndrome require specialist clinical input and patients should be managed as outpatients in secondary care.^29^

**Figure 3:**
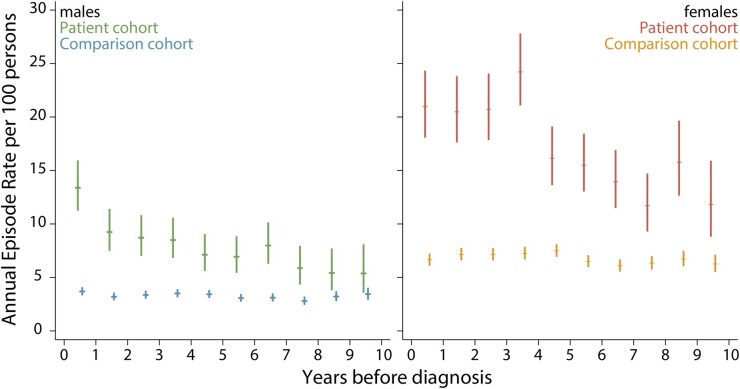
Annual rheumatology outpatient episode rates (95% confidence intervals) in the 10 years before diagnosis of diffuse large B-cell lymphoma (cases) or pseudo-diagnosis (controls): Haematological Malignancy Research Network diagnoses, 2009–2015

The utility of the comparison cohort to examine the relationship between rheumatological disorders and other morbidities is illustrated in Figure 3, which compares the annual rheumatology outpatient episode rates for male and female patients in the 10 years preceding their diagnosis of diffuse large B-cell lymphoma (DLBCL) to that of their general-population counterparts. Figure 3 clearly shows that female rates are around twice those of males (amongst DLBCL patients, as well as their comparators), with DLBCL patients being significantly more likely to have a history of rheumatoid disorders.^26^ Although the difference between males and females is as expected, it is notable that no evidence of effect modification by gender was found.

**Figure 4:**
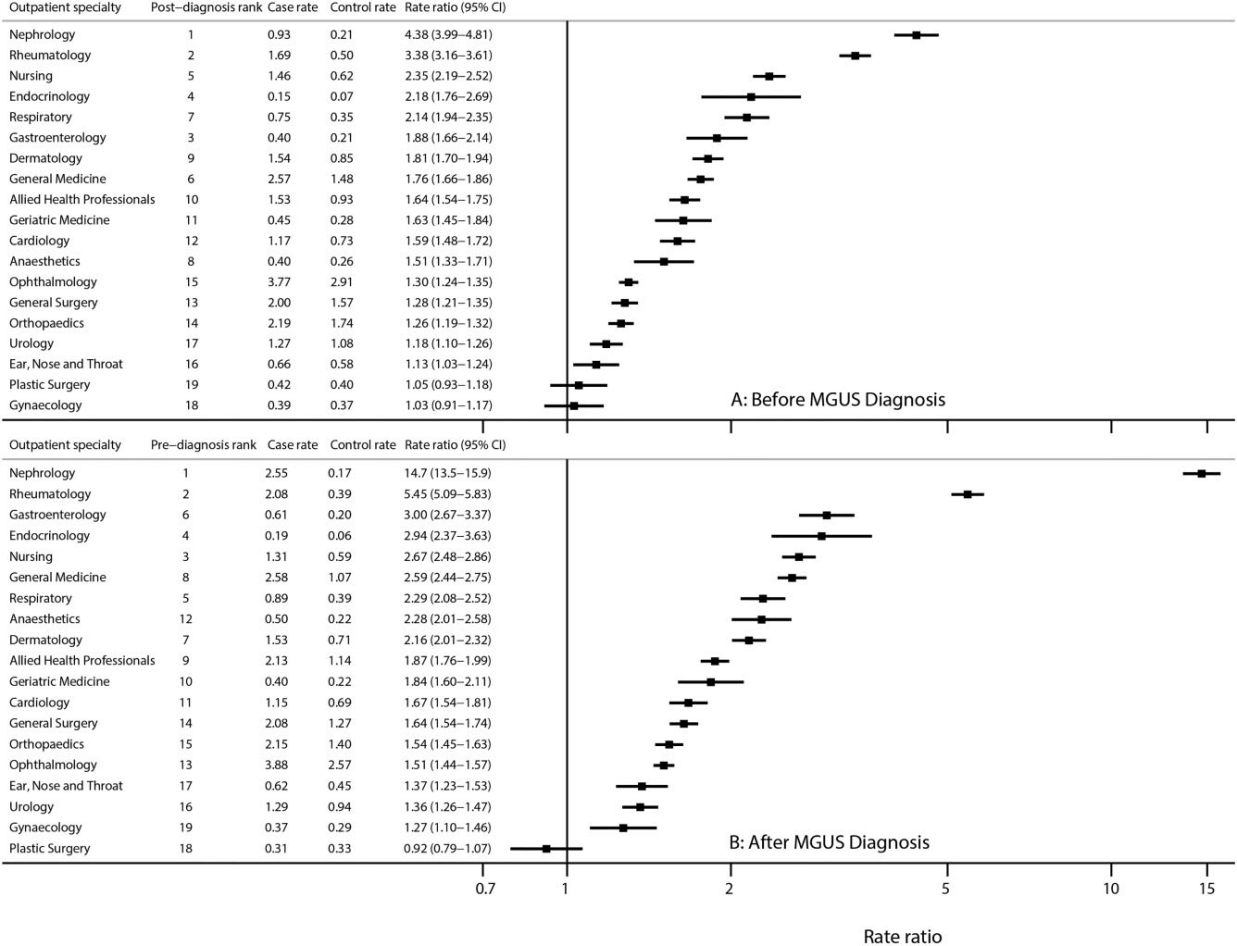
Monthly outpatient attendance rates (per 100 persons) in cases and controls, and rate ratios by outpatient specialty with at least two visits (A) in the 3 years before diagnosis and (B) in the 3 years after diagnosis of monoclonal gammopathy of undetermined significance (MGUS)

In addition to malignancies, HMRN also collects data on premalignancies (Figure 1). Here we were able to use the comparison cohort to provide information on background hospital attendance levels in the general population, finding that individuals with monoclonal gammopathy of undetermined significance (MGUS) experienced not only excess mortality and morbidity after diagnosis, but also excess morbidity in the 5 years before their premalignancy was diagnosed.^28^ By contrast, only marginal increases in mortality and morbidity were evident for monoclonal B-cell lymphocytosis (MBL), neither of which varied significantly from that of the general population. Figure 4 further illustrates the utility of our new comparison cohort data. Excluding haematology in which, as expected, outpatient attendances increased markedly just before and after MGUS diagnosis, the largest rate ratios (RRs) both before (Figure 4A) and after (Figure 4B) diagnosis were for nephrology [before diagnosis RR = 4.38, 95% confidence interval (CI) 3.99 to 4.81; after diagnosis RR = 14.7, 95% CI 13.5 to 15.9] and rheumatology (before diagnosis RR = 3.38, 95% CI 3.16 to 3.61; after diagnosis RR = 5.45, 95% CI 5.09 to 5.83). Other significant associations (*p* < 0.05) with RRs of >2.0 were evident for endocrinology, neurology and respiratory medicine, as well as for the nurse-led monitoring activities that form part of ongoing clinical care across a range of specialties.

## What are the main strengths and weaknesses?

Providing nationally generalizable data, major strengths of our population-based cohort include the large well-defined population, within which all haematological malignancies and related clonal disorders are diagnosed, monitored and coded using up-to-date standardized procedures. Based on world-class diagnostics, completeness of case ascertainment, adherence to national treatment guidelines and detailed follow-up of every patient, HMRN’s patient cohort is not affected by the data-quality issues faced by many population-based cancer registries. Predicated on infrastructures within the NHS, where universal healthcare is freely provided on the basis of clinical need, HMRN now occupies a unique forefront position in relation to the provision of information at key points along the patient pathway. With 10 individually age- and sex-matched controls for all patients diagnosed in 2009–2015, our new general-population cohort adds significant value to the original design, providing background rates and enabling us to examine associations with other co-morbidities and procedures, as well as healthcare activity patterns.

With respect to limitations, as noted in the original cohort report, although most haematological malignancies exhibit comparatively little geographic variation, a few are regionally very specific, e.g. endemic Burkitt lymphoma and adult T-cell leukaemia/lymphoma,^4^^,^^7^ both of which are extremely rare in our patient cohort. In addition, comparative analyses are currently confined to the information recorded in the sources listed in Figure 2; data from primary care, for example, are currently unavailable in the study region and, because of its pseudonymized nature, comparison cohort linkages to other data sets can only be performed by NHS Digital. Furthermore, whilst some information is well recorded in national data sets (e.g. deaths, cancer registrations and HES inpatient procedures and bed days), others are less reliable and/or incomplete (e.g. HES outpatient diagnoses and treatments). Finally, at present comparators are only available for patients diagnosed in 2009–2015, but having demonstrated the value of our approach, it is our intention to extend this when funding allows. 

## Can I get hold of the data? Where can I find out more?

Although ethical permissions and agreements with providers of national data mean that data deemed to have the potential to identify individuals cannot be transferred or accessed off-site, HMRN data are contributing to several ongoing research projects. For information on how to collaborate with HMRN researchers and investigate questions of interest, please e-mail enquiries (enquiries@hmrn.org) or e-mail the corresponding author (E.R.). Additional contact details are provided on the website (www.hmrn.org).

## Ethics approval

The HMRN has ethics approval (IRAS 289074) from Leeds West Ethics Committee, R&D approval from each NHS Trust and exemption from section 251 of the Health & Social Care Act (CAG 20/CAG/0149).

## Author contributions

E.R., E.K. and A.S. initiated the new data collection and E.R. drafted the manuscript. E.R., A.S. D.H., R.P. initiated the original cohort; A.S., E.K., M.L., T.B., S.C. and D.P. manage quantitative data and conduct analyses; and D.H. leads the qualitative work and Patient Public Involvement and Engagement (PPIE); and RP is the clinical lead, advising on the collection and interpretation of clinical data. All authors read and approved the final manuscript.

## Data availability

See ‘Can I get hold of the data?’ above.

## Funding

This work was supported by Cancer Research UK [grant numbers 18362 and 29685] and Blood Cancer UK [grant number 15037]. 

## Conflict of interest

None declared. 
